# Low calcium diet increases 4T1 mammary tumor carcinoma cell burden and bone pathology in mice

**DOI:** 10.1371/journal.pone.0180886

**Published:** 2017-07-27

**Authors:** Wendan Wang, Jody L. Gordon, Kenneth A. Philbrick, Xujuan Yang, Adam J. Branscum, Christiane V. Löhr, Wanda M. Haschek, Russell T. Turner, Urszula T. Iwaniec, William G. Helferich

**Affiliations:** 1 Food Science and Human Nutrition, University of Illinois, Urbana, Illinois, United States of America; 2 Skeletal Biology Laboratory, College of Public Health and Human Sciences, Oregon State University, Corvallis, Oregon, United States of America; 3 Biostatistics, College of Public Health and Human Sciences, Oregon State University, Corvallis, Oregon, United States of America; 4 Biomedical Sciences, College of Veterinary Medicine, Oregon State University, Corvallis, Oregon, United States of America; 5 Pathobiology, College of Veterinary Medicine, University of Illinois, Urbana, Illinois, United States of America; 6 Center for Healthy Aging Research, Oregon State University, Corvallis, Oregon, United States of America; Universite de Nantes, FRANCE

## Abstract

Breast cancer metastasizes to bone in the majority of patients with advanced disease. We investigated the effects of inadequate dietary calcium (Ca) on bone turnover, tumor growth, and bone response to tumor in tibia inoculated with 4T1 mammary carcinoma cells. Nine-month-old female Balb/c mice were placed on an adequate Ca (5 g/kg diet, n = 30) or low Ca (80 mg/kg diet, n = 31) diet for 14 days, then injected intratibially with 1,000 4T1 cells (transfected with luciferase for bioluminescence imaging), and sacrificed at 5, 10, or 21 days post-inoculation (n = 7–10 mice/group). Control mice (n = 6/group) were injected with carrier and sacrificed at 10 days post-inoculation. Tibiae with muscle intact were excised and evaluated by microcomputed tomography and histology. *In vivo* bioluminescent imaging revealed that 4T1 cells metastasized to lung. Therefore, lungs were removed for quantification of tumor. Mice fed low Ca exhibited higher bone turnover and higher tibial lesion scores than mice fed adequate Ca. Lesion severity, manifested as cortical osteolysis and periosteal woven bone formation, and tumor cell infiltration to muscle, increased with time, irrespective of diet. However, for most skeletal endpoints the rates of increase were greater in mice consuming low Ca compared to mice consuming adequate Ca. Infiltration of tumor cells into adjacent muscle, but not metastasis to lung, was also greater in mice consuming low Ca diet. The findings suggest that high bone turnover due to Ca insufficiency results in greater local mammary tumor cell growth, cortical osteolysis, woven bone formation, and invasion to muscle in mice.

## Introduction

Breast cancer metastasizes to bone in the majority of patients with advanced disease [1]. Skeletal morbidity includes chronic pain, hypercalcemia, pathologic fractures, and compression of the spinal cord and nerve roots [1]. One of these major complications occurs, on average, every 3 to 6 months, resulting in a severe reduction in the quality of life in individuals with breast cancer with metastasis to bone [2].

Breast cancer metastasis to bone involves an initial seeding of bone by cancer cells, subsequent tumor growth, and the potential for further metastasis to other sites. Metastases often appear initially as a single focus. Once metastasis has occurred, further metastasis to other bones and ultimately to soft tissues is common. Breast cancer preferentially metastasizes to vertebrae and long bone metaphyses [3, 4]. This distribution has not been fully explained, but one factor commonly discussed is the presence of an abundant vascular supply at the sites of metastases. However, the abundant blood supply co-localizes to sites where bone turnover rates are high. Bone turnover plays a key role in mineral homeostasis by supplying and withdrawing Ca to or from circulation, and is highly sensitive to Ca availability [5]. An elevated rate of bone turnover due to nutritional insufficiency may be an unsuspected important factor in breast cancer metastasis to bone and subsequent tumor growth.

The importance of the prevailing rate of bone turnover on tumor growth and metastasis has not been well established. We [6] and others [7] have shown that ovariectomy-induced increased bone turnover is associated with increased bone destruction in mouse models for mammary carcinoma metastasis to bone. Reduced estrogen status due to ovariectomy was unlikely to play a direct role in the tumor-mediated bone destruction as the cells we used to induce cancer (4T1 mammary carcinoma cells) were estrogen receptor negative. Dietary Ca insufficiency is well recognized as a cause of increased bone turnover and bone loss [8]. The purpose of the present study was to determine the effect of inadequate dietary Ca intake-induced increase in bone turnover on 4T1 mammary carcinoma cell burden and tissue (bone, muscle, and lung) response to tumor in mice inoculated with tumor cells in tibia.

## Materials and methods

Skeletally mature (9-month-old) female Balb/c mice were used in the experiment. The mice were obtained from the National Cancer Institute (Bethesda, MD) and housed individually in a temperature (21–23°C) and light (12 hr light/dark cycle) controlled room. Food and water were provided *ad libitum* to all animals. The mice were maintained in accordance with the NIH Guide for the Care and the Use of Laboratory Animals and the experimental protocol was approved by the Institutional Animal Care and Use Committee at the University of Illinois where the study was conducted.

### Experimental design

**Fig 1:**
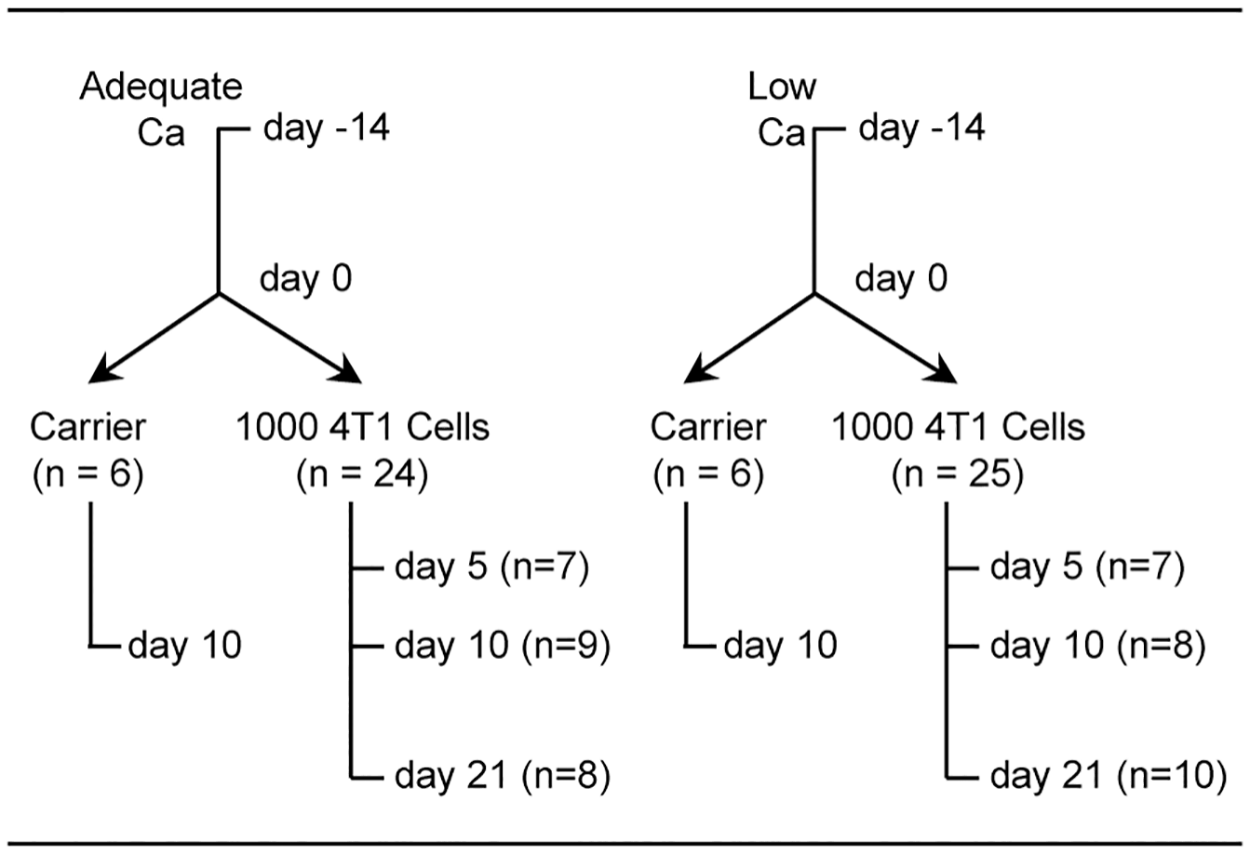
Experimental design. Skeletally mature (9-month-old) female Balb/c mice were randomized into one of 2 treatment groups: adequate Ca (5 g/kg diet) or low Ca (80 mg/kg diet) and maintained on their respective diets for 14 days to induce a state of increased bone turnover in the low Ca group. Mice within each dietary treatment were then subdivided into 4 groups. Three groups were injected in the tibia with 1,000 4T1 cells and sacrificed at 5, 10, or 21 days post 4T1 cell injection. Control mice were injected with carrier (Matrigel) and sacrificed at 10 days post carrier injection.

Sixty-one mice were randomized by weight into one of 2 dietary treatment groups (Fig 1): adequate Ca (5 g/kg diet, n = 30) or low Ca (80 mg/kg diet, n = 31) and maintained on their respective diets for 14 days to induce a state of increased bone turnover in the low Ca group prior to tumor injection. Mice within each diet group were then subdivided into 4 groups. Three groups (n = 7–10 mice/group) within each dietary treatment were injected in the right tibia with 1,000 4T1 cells tagged with firefly luciferase and sacrificed at 5, 10, or 21 days post 4T1 cell injection. Control mice (n = 6 mice/group) were injected with carrier (matrigel) and sacrificed at 10 days post injection. Each mouse was sequentially imaged on days 7, 11, 17 and 20 using bioluminescence. The fluorochrome calcein (20 mg/kg, Sigma, St Louis, MO) was administered at 4 days and 1 day prior to necropsy to label mineralizing bone. Tumor burden in tibia, muscle, and lung were quantified *ex vivo*. Tumor diameter in tibia was used as an approximation of tumor size. The largest tumor diameter measured in each mouse at 21 days on transverse histologic sections of the tibia was 5.4 +/- 0.4 cm for the normal calcium group and 6.2 +/- 0.5 cm for the low calcium group.

### Preparation of luciferase expressing 4T1 cells

Murine 4T1 mammary cancer cells tagged with firefly luciferase were obtained from Dr. David Piwnica-Worms (Washington University, St. Louis, MO). The cells were cultured in IMEM, supplemented with 10% HI-FBS, 100 unit/mL Penicillin, 100 μg/mL Streptomycin, 1% L-Glutamine, and 0.1% Fungizone in a humidified incubator containing 5% CO_2_ at 37°C. Cells were harvested with trypsin at 70% confluence, centrifuged (0°C, 700 rpm, 5 minutes), and suspended in Matrigel Basement Membrane Matrix (BD Biosciences, San Jose, CA) for injection.

### Intratibial injection of 4T1 cells

For intratibial 4T1 cell injection, mice were anesthetized with 2–3% isofluorane delivered in oxygen. The skin above the right knee was incised and a Hamilton syringe with a 26-gauge needle was inserted into the proximal tibia through the patellar tendon to a depth of 3 mm. The 26-gauge needle was replaced with a 27-gauge needle and 1,000 4T1 cells in 2.5 μl of Matrigel were injected into the tibia. The skin incision was sealed with adhesive (3M Vetbond, No. 1469SB) and closed with a surgical staple.

### Bioluminescence imaging

Tumor growth in bone and metastasis to lung were monitored using bioluminescence imaging. Mice were anesthetized with 2–3% isoflurane delivered in oxygen. Three minutes prior to imaging each mouse was injected with 15 mg/mL luciferin in PBS at a dose of 10 μL luciferin/g of body weight. Images were acquired with Piper Control software (Stanford Photonics, Palo Alto, CA) and analyzed using Image J (NIH, Bethesda, MD) and Photoshop Elements (Adobe, San Jose, CA). The background image of the mouse body was overlaid with the image showing the luminescence of the tumor.

### Tissue collection

For tissue collection, mice were anesthetized with CO_2_ gas and sacrificed by cervical dislocation. Tibiae with muscle intact were excised, placed in 10% phosphate-buffered formalin for 1 day and stored in 70% ethanol for analysis of tumor burden and bone response to tumor using microcomputed tomography (μCT) and histology. Lungs were perfused with India ink for assessment of tumor number in lung.

### Assessment of bone response to low Ca intake

#### μCT imaging

μCT was used for nondestructive 3-dimensional evaluation of cancellous and cortical bone response to Ca intake. Tibiae were scanned in 70% ethanol at a voxel size of 16 μm x 16 μm x 16 μm (55 kV_p_ x-ray voltage, 145 μA intensity, and 200 ms integration time) using a μCT40 scanner (Scanco Medical AG, Basserdorf, Switzerland). Filtering parameters sigma and support were set to 0.8 and 1, respectively. For assessment of cancellous bone, 20 slices (320 μm), 320 μm distal to the growth plate, were evaluated in the proximal tibial metaphysis at a threshold of 245 (scale 0–1000). The region of interest was delineated manually a few pixels away from the endocortical surface. Cancellous bone measurements included cancellous bone volume fraction (bone volume/tissue volume, %), trabecular number (mm^-1^), trabecular thickness (μm) and trabecular separation (μm). For assessment of cortical bone, 20 slices (320 μm) were evaluated in the tibial diaphysis 240 μm proximal to the tibio-fibular junction at a threshold of 245. Cross-sectional tissue volume (cortical and marrow volume, mm^3^), cortical volume (mm^3^), marrow volume (mm^3^), cortical thickness (μm), and polar moment on inertia (mm^4^), an index of bone strength in torsion, were measured.

#### Histology

Following μCT analysis, proximal tibiae were prepared for histomorphometric evaluation of bone turnover as described [9]. The bones were dehydrated in graded increases of ethanol and xylene and embedded undecalcified in methyl methacrylate. Four-μm-thick sections were cut with a vertical bed microtome (Leica 2165) and affixed to slides precoated with a 1% gelatin solution. Sections (1/mouse) were stained for tartrate resistant acid phosphatase and counterstained with toluidine blue (Sigma, St Louis, MO, USA) for measurement of osteoclast and osteoblast perimeters. Sections (1/mouse) were also mounted unstained for measurement of fluorochrome labels. Osteoblast perimeter was determined as a percentage of cancellous bone perimeter lined by a palisade of plump cuboidal cells located immediately adjacent to a thin layer of osteoid in direct physical contact with the bone perimeter (osteoblast perimeter/bone perimeter, %). Osteoclast perimeter was determined as the percentage of cancellous bone perimeter covered by multinucleated (two or more nuclei) cells with tartrate resistant acid phosphatase positive cytoplasm (osteoclast perimeter/bone perimeter, %). Fluorochrome-based measurements of bone formation included: (1) mineralizing perimeter (cancellous bone perimeter covered with double plus half single label divided by bone perimeter, %), (2) mineral apposition rate (the distance between two fluorochrome markers that comprise a double label divided by the 3 day interlabel interval, μm/day), and (3) bone formation rate (calculated by multiplying mineralizing perimeter by mineral apposition rate divided by bone perimeter, μm^2^/μm/yr). Histomorphometric data were collected using the OsteoMeasure System (OsteoMetrics, Inc., Atlanta, GA, USA) in a sampling site located 0.25–1.25 mm distal to the growth plate and 0.1 mm from cortical bone. Data are reported using standard 2-dimensional nomenclature [10].

### Assessment of tumor burden in tibia and muscle

Tumor burden and tissue response to tumor were evaluated using μCT and histology.

#### μCT imaging

4T1 cell-inoculated tibiae were scanned with muscle intact in 70% ethanol as described above. Bone response to tumor was evaluated using semi-quantitative (total tibia pathology score) and quantitative (measurement of bone) assays.

Total tibia pathology score. Total tibiae were imaged at a threshold of 245 (scale of 0–1000) and the reconstructed 3-dimensional images used for visual assessment of bone response to tumor. Bone response was characterized by pathological periosteal bone formation (woven bone extending from the periosteum) and cortical osteolysis (pathological bone resorption). The total bone response to tumor was scored on a scale from 0 (normal bone) to 5 (extensive woven bone/osteolysis).

Quantitative assessment of pathological periosteal woven bone and cortical osteolysis. In order to quantify the extent of periosteal woven bone and cortical osteolysis, a μCT image stack of 500 slices (8 mm of bone) proximal to tibiofibular junction was analyzed. Woven bone was distinguished from cortical bone based on μCT voxel threshold. All voxels residing within 64 μm of the cortical bone surface were excluded from analysis to remove partial volume effects. Pathological woven bone was defined by voxels with threshold values ranging from 150–424 (scale 0–1000). Cortical bone was defined as voxels with thresholds of 523–1000.

#### Histology

Following μCT analysis, tibiae with muscle intact were decalcified and embedded in paraffin. The bones were cut transversely at the tibiofibular junction and at 4, 6, 8 and 10 mm proximal to the tibiofibular junction and stained with hematoxylin and eosin.

Histopathology score. Tissue response to tumor was evaluated separately in (1) medullary cavity (bone marrow), (2) cortical bone, (3) periosteal surface, and (4) muscle. Tumor cells were inserted proximal to the sites evaluated. The analysis for each anatomical location was performed on 5 histological sections (tibiofibular junction and at 4, 6, 8 and 10 mm proximal to the tibiofibular junction) using a 0–5 scoring system. For the medullary cavity, a score of 0 represents normal histology without the presence of tumor cells whereas scores of 1 to 5 represent progressively increasing levels of infiltration of tumor cells into bone marrow and subsequent bone marrow cell necrosis. Thus, increasing score is assumed to reflect an increase in tumor burden. For cortical bone, a score of 0 represents normal histology whereas scores of 1 to 5 represent progressively increasing levels of pathological endocortical and/or periosteal resorption (osteolysis). For the cortical periosteum, a score of 0 represents normal histology whereas scores of 1 to 5 represent progressively increasing levels of pathological woven bone formation extending from the periosteum into adjacent tissue. For muscle, a score of 0 represents normal muscle whereas scores of 1 to 5 represent progressively increasing levels of infiltration of tumor cells into muscle.

### Assessment of tumor burden in lung

Lungs of mice were perfused with India ink for tumor visualization [6]. Briefly, ribs were cut to expose lung and trachea, and India ink was slowly infused into the lung via the trachea until the lung was fully expanded. The lungs were then harvested and fixed in Fekete solution. Following transfer to fresh Fekete solution for 24 hours, tumors on lung lobes were counted. Tumors were identified as white (unstained) nodules (1–2 mm in diameter) in black lung tissue.

### Statistical analysis

Cancellous bone architecture and turnover in the proximal tibia metaphysis and cortical bone architecture in the distal tibia diaphysis were compared between the adequate and low Ca groups using paired t-tests, independent two-sample t-tests or Wilcoxon nonparametric tests. Comparisons of the adequate and low Ca groups were made at 5, 10, and 21 days post injection of 4T1 cancer cells using t-tests, Wilcoxon tests, two-way ANOVA with interaction, and multiple linear regression to evaluate time trend. The required conditions for valid use of Gaussian linear models were assessed using Levene’s test for homogeneity of variance, plots of residuals versus fitted values, normal quantile plots, and the Anderson-Darling test of normality. The Benjamini and Hochberg method for maintaining the false discovery rate at 5% was used to adjust for multiple comparisons [11]. Data analysis was performed using R version 3.3.2 [12].

## Results

### Low Ca diet resulted in higher cancellous bone turnover in tibia

The effects of 24 days of low Ca intake on cancellous and cortical bone architecture (μCT) and on cancellous bone turnover (histomorphometry) in proximal tibia metaphysis are shown in Table 1. Cancellous bone volume fraction and trabecular thickness were lower and trabecular separation tended to be higher (P = 0.063) in mice consuming low Ca compared to mice consuming adequate Ca diet. Significant differences in trabecular number were not detected with treatment. Osteoclast perimeter, osteoblast perimeter, mineralizing perimeter, and bone formation rate were all higher and mineral apposition rate tended to be higher (P = 0.063) in mice consuming low Ca diet. Significant differences in tibial diaphysis cross-sectional volume, cortical volume, marrow volume, cortical thickness, or polar moment of inertia were not detected with treatment.

**Table 1:** Effects of 24 days of low Ca intake on cancellous bone architecture and turnover in proximal tibia metaphysis and on cortical bone architecture in distal tibia diaphysis.

	Adequate Ca	Low Ca	FDR-adjusted P-value
**Proximal Tibia Metaphysis** (cancellous bone)			
***Architecture (μCT)***			
Bone volume/tissue volume (%)	29.8 ± 2.1	12.9 ± 1.1	**0.001**
Trabecular number (mm^-1^)	7.2 ± 0.1	7.2 ± 0.1	0.975
Trabecular thickness (μm)	65 ± 2	50 ± 1	**0.001**
Trabecular separation (μm)	154 ± 7	170 ± 2	0.063
***Turnover (histomorphometry)***			
Osteoclast perimeter/bone perimeter (%)	4.3 *±* 1.3	15.6 *±* 1.6	**0.002**
Osteoblast perimeter/bone perimeter (%)	7.3 *±* 1.9	26.9 *±* 3.9	**0.007**
Mineralizing perimeter/bone perimeter (%)	7.7 *±* 2.2	25.2 *±* 4.2	**0.018**
Mineral apposition rate (μm/d)	1.1 *±* 0.2	1.5 *±* 0.1	0.063
Bone formation rate/bone perimeter (μm^2^/μm/y)	36.3 *±* 12.2	133.8 *±* 18.5	**0.007**
**Distal Tibia Diaphysis** (cortical bone)			
***Architecture (μCT)***			
Cross-sectional volume (mm^3^)	0.282 *±* 0.006	0.285 *±* 0.005	0.838
Cortical volume (mm^3^)	0.237 *±* 0.005	0.233 *±* 0.004	0.699
Marrow volume (mm^3^)	0.046 *±* 0.004	0.053 *±* 0.002	0.121
Cortical thickness (μm)	304 *±* 8	294 *±* 2	0.391
Polar moment of inertia (mm^4^)	0.121 *±* 0.005	0.122 *±* 0.005	0.975

Data are mean ± SE, n = 6/group

To determine whether a low Ca diet resulted in progressive bone loss, we evaluated the effects of Ca intake on the contralateral (uninjected) tibia in 4T1 cell injected mice sacrificed 5, 10 and 21 days post cell injection (19, 24 and 31 days following onset of low Ca diet, respectively) (S1 Table**)**. Similar cancellous osteopenia (lower bone volume fraction and trabeculae thickness and higher trabecular separation) was evident in mice fed low Ca diet at all 3 time points. Additionally, low Ca diet resulted in lower marrow volume and lower cortical thickness.

We also determined the effect of inserting a needle for delivery of 4T1 cells into the proximal tibia on bone microarchitecture. μCT analysis was performed in control mice sacrificed 10 days following needle insertion. Representative images for intact and needle injected tibia are shown in S1 Fig. The needle track is visible in the injected limb in adequate Ca and low Ca mice and bone healing is evident in both treatment groups. Quantification of the bone response to needle insertion in tibia is presented in S2 Table. Significant differences in cancellous bone architecture between the uninjected (left) and injected (right) bones were not detected in mice fed adequate Ca. In contrast, cancellous bone volume fraction was higher, trabecular number and thickness tended to be higher (P = 0.10 and 0.081, respectively), and trabecular separation was lower in the injected (right) compared to the uninjected (left) tibia in mice consuming low Ca diet. However, a test for an interaction revealed no difference between the adequate and low Ca groups, indicating a lack of difference in skeletal response to needle injury.

### 4T1 tumor burden in tibia and adjacent muscle and tibial response to tumor were greater in mice fed low Ca diet

#### Histology

**Fig 2:**
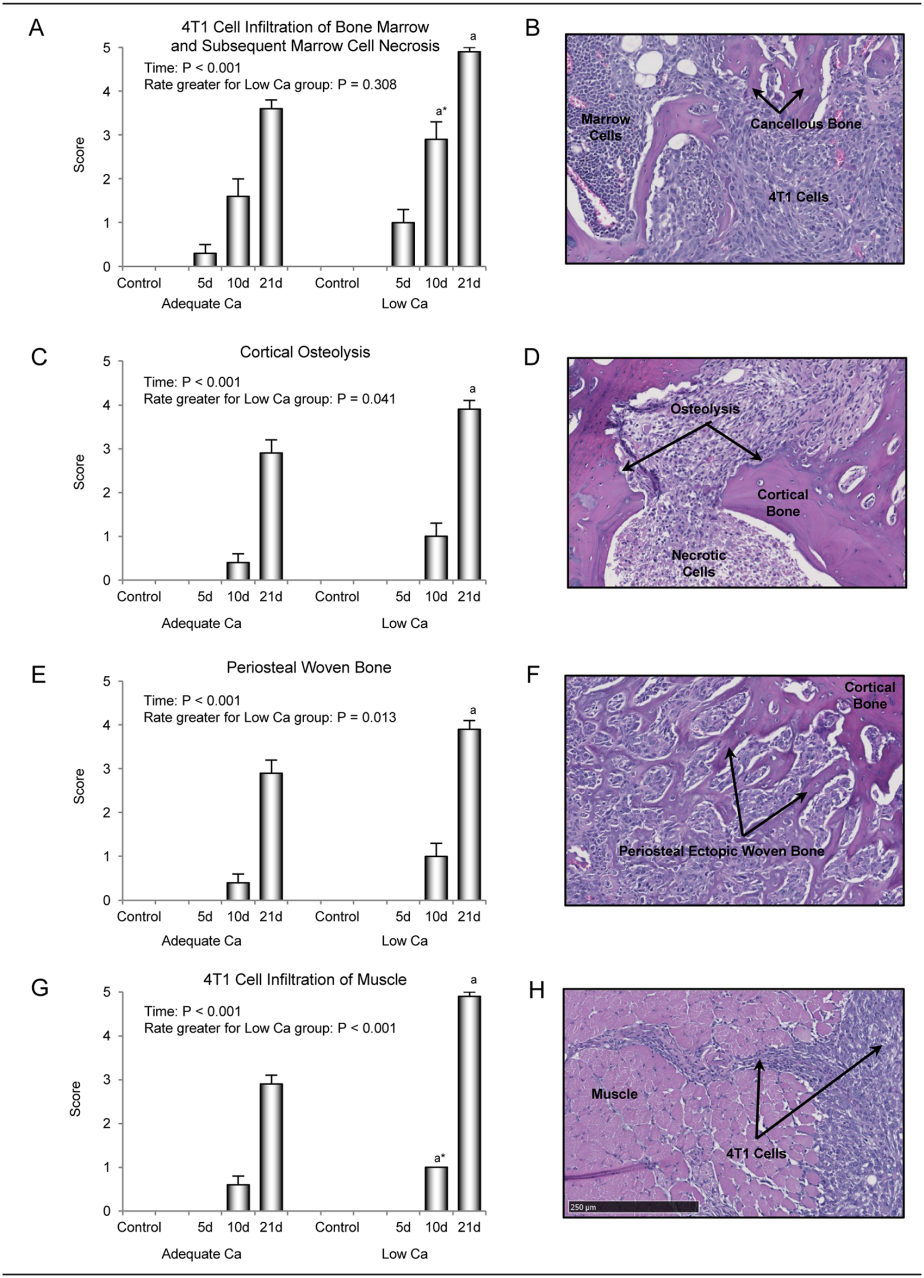
Histological evaluation of the effects of low Ca intake on tumor infiltration of bone marrow (A and B), cortical osteolysis (C and D), periosteal woven bone formation (E and F), and tumor infiltration of muscle (G and H) in the tibial diaphysis at 5, 10, and 21 days following intratibial 4T1 mammary carcinoma cell injection. Histopathology (assessed visually and scored from 0 to 5) increased with time for each of the endpoints evaluated (A, C, E, and G), but the rate of increase in cortical osteolysis (B), periosteal woven bone formation (E), and tumor infiltration of muscle (G) was greater in mice fed low Ca diet. Tissue response to tumor was greater in the low Ca compared to the adequate Ca group on day 21 for all endpoints evaluated. Representative photomicrographs illustrate the extensive tumor infiltration of bone marrow (B), cortical osteolysis (D), periosteal woven bone formation (F), and tumor infiltration of muscle (H) following 4T1 cell injection in mice fed the low Ca diet. Data are mean ± SE. ^a^Different from adequate Ca group on same day, P < 0.05, ^a^* P < 0.1.

Time-dependent changes in tibia and adjacent muscle in response to intratibial 4T1 mammary carcinoma cell injection were evaluated by histology. The relationship between Ca intake and tumor in marrow (infiltration of tumor cells into marrow cavity and subsequent bone marrow cell necrosis) and accompanying histopathology in cortex (cortical osteolysis resulting in fragmentation of cortical bone), periosteum (periosteal woven bone formation), and muscle (infiltration of tumor cells into muscle) at 5, 10, and 21 days following intratibial 4T1 cell injection are shown in Fig 2. Tissue response to tumor cell injection increased with time for all endpoints evaluated (Fig 2A, 2C, 2E and 2G). However, the rate of increase (slope) in cortical osteolysis (Fig 2C), periosteal woven bone formation (Fig 2E), and tumor cell infiltration into muscle (Fig 2G) was greater in mice consuming low Ca compared to mice consuming adequate Ca diet. Paired analysis at each time point (5, 10, and 21 days post cell injection) was also performed. Treatment differences were not detected between mice consuming low Ca and adequate Ca diets at either 5 or 10 days following cell injection. However, there was a tendency (P = 0.076) for tumor cell infiltration into marrow (Fig 2A) and muscle (Fig 2G) to be greater in mice consuming the low Ca diet at 10 days post cell injection. Cortical osteolysis, periosteal woven bone formation, and tumor infiltration into muscle were greater at 21 days post cell injection in mice consuming low Ca compared to mice consuming adequate Ca diet. On day 21, marrow was primarily necrotic in mice consuming low Ca diet.

#### Microcomputed tomography

**Fig 3:**
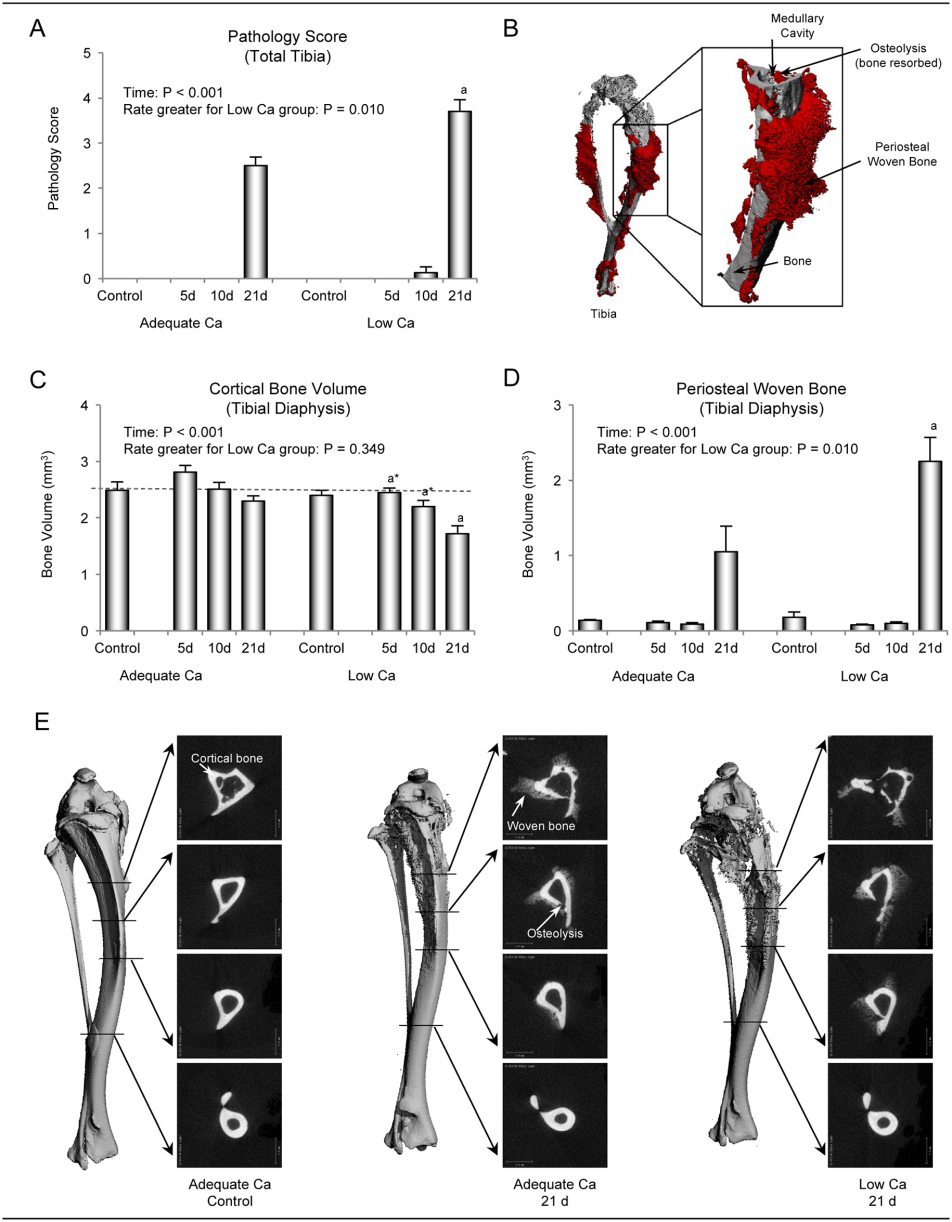
μCT evaluation of the effects of low Ca intake on bone response to tumor in the tibial diaphysis 5, 10, and 21 days following intratibial 4T1 mammary carcinoma cell injection. Total tibia pathology (assessed visually and scored from 0 to 5) increased with time, but the rate of increase was greater in mice fed low Ca compared to mice fed adequate Ca (A). Following visual evaluation, cortical osteolysis and periosteal woven bone were quantified based on differential thresholding (B). Cortical bone volume decreased with time, indicative of increased osteolysis (C). Although the rates of decrease did not differ between mice fed adequate and mice fed low Ca diets, cortical bone volume was lower in mice fed low Ca compared to mice fed adequate Ca at 21 days post 4T1 cell injection. Periosteal woven bone formation increased with time and the rate of increase was greater in mice fed low Ca diet (D). Representative μCT images of tibia from a control mouse and mice administered 4T1 cells fed either adequate or low Ca diet and sacrificed at 21 days post 4T1 cell injection are shown in E. The images illustrate the distribution of cortical osteolysis and periosteal woven bone. Data are mean ± SE. ^a^Different from adequate Ca group on same day, P < 0.05, ^a^* P < 0.1.

Time-dependent tibial response to tumor was independently evaluated using μCT (Fig 3). The extent of pathological periosteal woven bone and cortical osteolysis in total tibia (score based on visual assessment) increased with time (Fig 3A). However, the rate of increase was greater in mice consuming low Ca compared to mice consuming adequate Ca diet. Treatment differences were not detected between mice consuming low and adequate Ca diets at either 5 or 10 days following tumor cell injection, but pathology score was higher at 21 days post tumor cell injection in mice consuming low Ca.

Bone response to tumor in tibial diaphysis was subsequently quantified by μCT measurement of (1) cortical bone osteolysis and (2) periosteal woven bone (Fig 3B). Cortical bone volume decreased with time in response to tumor (Fig 3C). However, a significant difference in the rates of decrease was not detected with Ca intake. Cortical bone volume tended to be lower at 5 (P = 0.072) and 10 (P = 0.093) days and was lower at 21 days following 4T1 cell injection in mice consuming low Ca compared to time-matched mice fed adequate Ca diet. Periosteal woven bone formation in response to tumor increased with time (Fig 3D), and the slope for the increase was steeper in mice consuming low Ca compared to mice consuming adequate Ca. Differences were not detected between the treatment groups at either 5 or 10 days following cell injection but the bone response was greater at 21 days post tumor cell injection in mice consuming low Ca diet. Bone response to treatment on day 21 can be readily appreciated in Fig 3E.

### Metastatic progression of 4T1 cells to lung was not impacted by dietary Ca status

**Fig 4:**
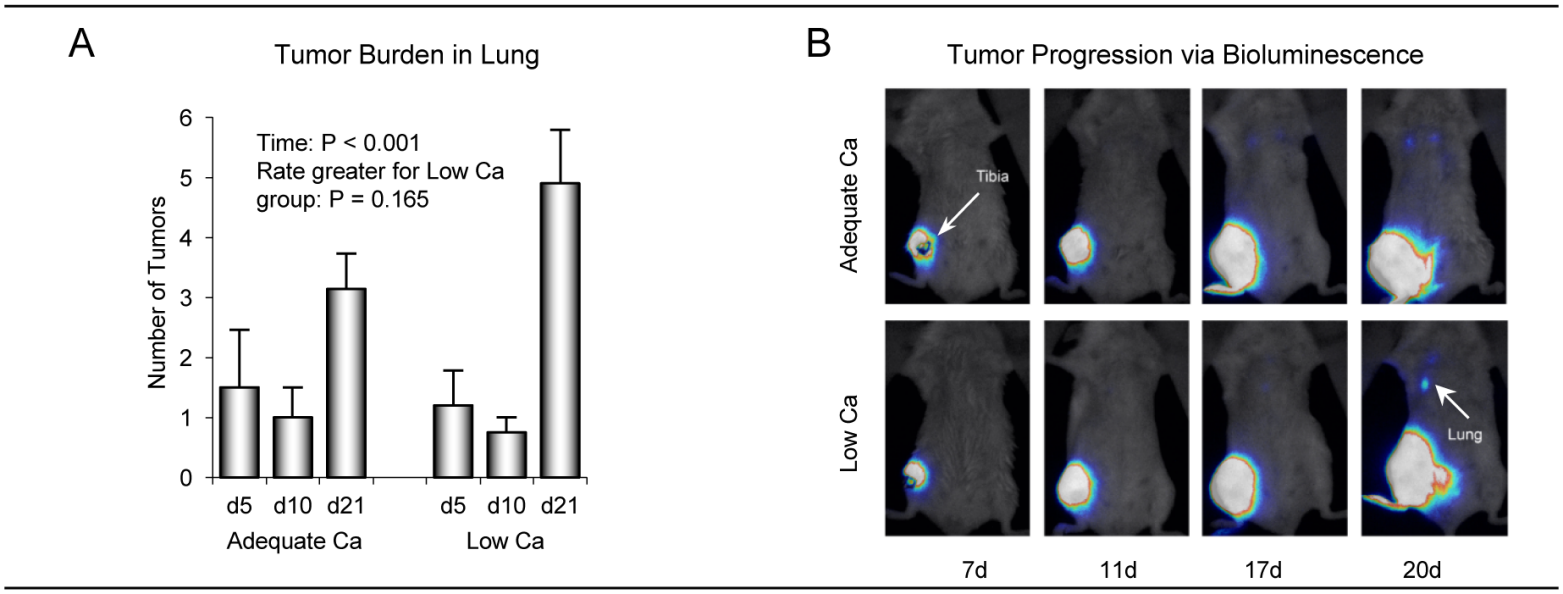
Effects of low Ca intake on metastatic progression of 4T1 mammary carcinoma cells to lung 5, 10, and 21 days following intratibial 4T1 cell injection. Tumor number in lung increased with time independent of Ca intake (A). Bioluminescence images from 2 representative mice at 7, 11, 17 and 20 days following intratibial 4T1 cell injection (B). Data are mean ± SE.

The relationship between Ca intake and metastatic tumor burden in lung at 5, 10, and 21 days following intratibial 4T1 cell injection are shown in Fig 4. Tumor number in lung increased with time (Fig 4A). However, a significant difference in the rates of increase was not detected with Ca intake. Furthermore, treatment differences were not detected between mice consuming low and adequate Ca at either 5, 10, or 21 days following tumor cell injection. Representative images of metastatic progression of 4T1 cells to lung using bioluminescence at 7, 11, 17, and 20 days following intratibial 4T1 cell injection are shown in Fig 4B.

## Discussion

Feeding mice low Ca diet resulted in increased bone turnover and cancellous bone loss. Inoculation of tibia with 4T1 breast carcinoma cells resulted in cortical osteolysis, periosteal woven bone formation, tumor cell infiltration into adjacent muscle, and metastasis to lung. Tumor burden in bone marrow and bone response to tumor (cortical osteolysis and periosteal woven bone formation) were increased in mice fed a low Ca diet. Infiltration of 4T1 cells into adjacent muscle was also increased in mice fed low Ca diet. In contrast, we did not detect diet-induced differences in metastasis to lung.

The 4T1 mammary carcinoma transplantable tumor cell line is highly tumorigenic and invasive and has been shown to metastasize from the primary tumor in the mammary gland to multiple distant sites including lymph nodes, blood, liver, lung, brain, and bone [13]. Importantly, micrometastatic breast cancer cells are found in bone of up to 80% of patients who died from the disease. Micrometastatic breast cancer cells can stay dormant in bone for years but when activated lead to bone destruction [14–16], inducing higher risk of fracture, hypercalcemia, bone pain, and paralysis due to spinal cord compression [17, 18].

Braun et al. reported that 30% of breast cancer patients had bone micrometastasis at diagnosis of breast cancer [16]. Furthermore, many of these patients had estrogen receptor-negative tumors. 4T1 cells are triple negative (estrogen receptor negative, progesterone receptor negative, human epidermal growth factor receptor 2 negative). As a consequence, 4T1 cells grow *in vitro* and in mice in the absence of estrogen. We recently demonstrated that ovariectomy, a model for increased bone turnover following menopause, increases pathological bone response to tumor in tibia of mice inoculated with 4T1 cells [6]. This finding suggests that menopause has the potential to activate and/or enhance the growth of triple negative breast cancer cells within the bone marrow environment.

Breast carcinoma-induced osteolysis is mediated by osteoclasts whose differentiation is stimulated, at least in part, by local production of the tumor cell-derived parathyroid hormone-related peptide (PTH-rP). Production of PTH-rP by breast carcinoma cells in bone is enhanced by growth factors, such as transforming growth factor-beta (TGF-β) and insulin like growth factor-1 (IGF-1), originally generated by osteoblasts, deposited into bone matrix as inactive growth factor-binding protein complexes, and subsequently activated upon release from bone matrix by osteoclast-mediated bone resorption [1]. Thus, osteoblast-generated growth factors enhance PTH-rP production, creating a vicious cycle where bone resorption and tumor growth reciprocally enhance each another [1]. Additionally, osteoblast lineage cells regulate bone resorption via regulation of the osteoclast differentiation factor RANKL. Bone resorption has been reported to be essential to growth of estrogen receptor negative breast cancer cells in bone but not in fat pad [19, 20]. This finding may be relevant to our finding of increased tumor burden in bone but not lung in mice fed a low Ca diet.

Recent studies have identified hypoxic induction of lysyl oxidase with metastatic dissemination to bone in estrogen receptor-negative breast cancer [21]. Lysyl oxidase catalyzes cross-linking of elastin and collagen and has a variety of non-enzymatic regulatory actions. Expression of lysyl oxidase is often upregulated in hypoxic breast tumors and indeed appears to be required for tumor growth and metastasis [22]. Importantly, suboptimal Ca intake and impaired Ca absorption are common following menopause, resulting in elevated parathyroid hormone (PTH) levels [23, 24]. Elevated PTH levels, in turn, result in increased lysyl oxidase expression in skeletal tissue in rodents [25]. Thus, there are multiple mediators that may drive tumor growth in bone independent of growth in other tissues.

Dietary Ca has the potential to impact cancer risk and progression. Some, but not all, epidemiological studies have linked low Ca intake to increased breast cancer risk [26]. Few studies have investigated the impact of dietary Ca on breast cancer progression in women [27]. However, low dietary Ca was reported by Zheng et al., to accelerate breast cancer tumor growth in bone in mice [28]. Specifically, tumor cell proliferation, tumor area, and lytic lesion area were increased in Ca-deficient nude mice inoculated with Tx-SA cells derived from MDA-MB-231 cells. The present study confirms these findings using the syngeneic 4T1 mammary tumor cell model in Balb/c mice.

In addition, the present work extends Zheng et al., [28] by evaluating the impact of low dietary Ca on periosteal woven bone formation and metastasis concurrent with mammary tumor growth within bone marrow. Mammary tumors that metastasize to bone can be lytic, sclerotic or mixed (lytic and sclerotic), with the latter being most common [29]. In spite of Ca deficiency, cancellous bone formation was elevated in tumor-free control mice fed the low Ca diet. Similarly, periosteal woven bone formation in response to 4T1 cell inoculation was greater in the Ca-deficient mice. Thus, normal as well as tumor-driven bone formation are increased in mice fed low Ca diet.

The increased tumor growth and bone destruction associated with a Ca-deficient diet reported by Zheng et al., was attributed to increased bone resorption [28]. The present study demonstrates that bone formation as well as bone resorption were increased, indicating a general increase in bone turnover. Ca deficiency, however, could have additional contributing effects. Rodents fed a low Ca diet develop secondary hyperparathyroidism [30]. PTH receptors are frequently expressed in mammary cancers that have metastasized to bone and signaling through these receptors is believed to drive cancer cell proliferation as well as increase bone turnover [31]. An important PTH-independent role of increased bone turnover in the lytic response to 4T1 cells is illustrated in our recent study in ovariectomized mice [6]. Specifically, ovariectomy was shown to increase tibial destruction following inoculation of 4T1 cells, in spite of 4T1 cells being ER negative. Ovariectomy results in an increase in bone turnover in rodents but does not result in secondary hyperparathyroidism [32]. Taken together, these findings suggest that high bone turnover rather than Ca deficiency or estrogen deficiency is responsible, at least in part, for the increased bone response to tumor.

Skeletal muscle metastasis is generally uncommon and usually indicates an advanced disease associated with poor prognosis [33]. Breast cancer metastasis to muscle has been shown to occur in women [34, 35]. The MDA-MB-435 human breast cancer cell line metastasizes from tumors growing in the mammary fat pad of nude mice; metastases were found in the lymph nodes and lungs of the majority of tumor-bearing mice, and at a lower incidence in other organs such as the heart, adrenal gland, brain, and skeletal muscle [36]. In the present study, tumor cell infiltration into adjacent muscle was observed initially on day 10 in some mice and in all mice at 21 days following tumor inoculation into tibia. Similar to 4T1 cell infiltration of bone marrow, cortical osteolysis, and periosteal woven bone formation, tumor burden in muscle was greater in Ca-deficient mice.

In summary, elevated bone turnover, induced by a low Ca diet, was associated with increased local tumor burden in a murine model for bone micrometastasis. Additionally, the low Ca diet resulted in increases in osteolysis, periosteal woven bone formation, and infiltration to adjacent muscle. These findings further implicate elevated bone turnover as a factor that increases late stage mammary tumor growth and progression.

## Supporting information

S1 Table Effects of Ca intake on cancellous and cortical bone architecture in contralateral (left; uninjected) proximal tibia metaphysis and distal tibia diaphysis, respectively in mice injected with 4T1 cells in right tibia and sacrificed on day 5, 10, and 21 post 4T1 cell injection. (PDF)

S2 Table Effects of Ca intake and needle insertion on cancellous and cortical bone microarchitecture in tibia at 10 days post carrier injection. (PDF)

S1 Fig Representative CT images of proximal tibia metaphysis showing the residual needle insertion track (injection site) in a mouse fed adequate Ca diet and a mouse fed low Ca diet. Note the presence of bone spicules in the needle track of both treatment groups.
(TIF)

## References

[1] Mundy GR. Mechanisms of bone metastasis. Cancer. 1997;80(8 Suppl):1546–56. . 9362421 10.1002/(sici)1097-0142(19971015)80:8+<1546::aid-cncr4>3.3.co;2-r

[2] Coleman RE. Clinical features of metastatic bone disease and risk of skeletal morbidity. Clin Cancer Res. 2006;12(20 Pt 2):6243s–9s. doi: 10.1158/1078-0432.CCR-06-0931 . 17062708 10.1158/1078-0432.CCR-06-0931

[3] Boxer DI, Todd CE, Coleman R, Fogelman I. Bone secondaries in breast cancer: the solitary metastasis. J Nucl Med. 1989;30(8):1318–20. . 2754488

[4] Krishnamurthy GT, Tubis M, Hiss J, Blahd WH. Distribution pattern of metastatic bone disease. A need for total body skeletal image. JAMA. 1977;237(23):2504–6. . 576963

[5] Reid IR, Ames RW, Evans MC, Gamble GD, Sharpe SJ. Effect of calcium supplementation on bone loss in postmenopausal women. N Engl J Med. 1993;328(7):460–4. doi: 10.1056/NEJM199302183280702 . 8421475 10.1056/NEJM199302183280702

[6] Wang W, Belosay A, Yang X, Hartman JA, Song H, Iwaniec UT, et al Effects of letrozole on breast cancer micro-metastatic tumor growth in bone and lung in mice inoculated with murine 4T1 cells. Clin Exp Metastasis. 2016;33(5):475–85. doi: 10.1007/s10585-016-9792-z . 27209469 10.1007/s10585-016-9792-z

[7] Ottewell PD, Wang N, Brown HK, Fowles CA, Croucher PI, Eaton CL, et al OPG-Fc inhibits ovariectomy-induced growth of disseminated breast cancer cells in bone. International journal of cancer. 2015;137(4):968–77. doi: 10.1002/ijc.29439 . 25603921 10.1002/ijc.29439

[8] Nordin BE. Calcium and osteoporosis. Nutrition. 1997;13(7–8):664–86. . 9263260 10.1016/s0899-9007(97)83011-0

[9] Iwaniec UT, Wronski TJ, Turner RT. Histological analysis of bone. Methods Mol Biol. 2008;447:325–41. doi: 10.1007/978-1-59745-242-7_21 . 18369927 10.1007/978-1-59745-242-7_21

[10] Dempster DW, Compston JE, Drezner MK, Glorieux FH, Kanis JA, Malluche H, et al Standardized nomenclature, symbols, and units for bone histomorphometry: a 2012 update of the report of the ASBMR Histomorphometry Nomenclature Committee. J Bone Miner Res. 2013;28(1):2–17. doi: 10.1002/jbmr.1805 ; 23197339 10.1002/jbmr.1805 PMC3672237

[11] Benjamini Y, Hochberg Y. Controlling the false discory rate: a practical and powerful approach to multiple testing. J Royal Statistical Society Series B. 1995;57:289–300.

[12] Team RC. R: A language and environment for statistical computing Vienna, Austria: R Foundation for Statistical Computing; 2015.

[13] Pulaski BA, Ostrand-Rosenberg S. Mouse 4T1 breast tumor model. Curr Protoc Immunol. 2001;Chapter 20:Unit 20 2 doi: 10.1002/0471142735.im2002s39 . 18432775 10.1002/0471142735.im2002s39

[14] Chen YC, Prabhu KS, Das A, Mastro AM. Dietary selenium supplementation modifies breast tumor growth and metastasis. Int J Cancer. 2013;133(9):2054–64. doi: 10.1002/ijc.28224 . 23613334 10.1002/ijc.28224

[15] Tester AM, Ruangpanit N, Anderson RL, Thompson EW. MMP-9 secretion and MMP-2 activation distinguish invasive and metastatic sublines of a mouse mammary carcinoma system showing epithelial-mesenchymal transition traits. Clin Exp Metastasis. 2000;18(7):553–60. . 11688960 10.1023/a:1011953118186

[16] Braun S, Vogl FD, Naume B, Janni W, Osborne MP, Coombes RC, et al A pooled analysis of bone marrow micrometastasis in breast cancer. N Engl J Med. 2005;353(8):793–802. doi: 10.1056/NEJMoa050434 . 16120859 10.1056/NEJMoa050434

[17] Guise TA. Breast cancer bone metastases: it's all about the neighborhood. Cell. 2013;154(5):957–9. doi: 10.1016/j.cell.2013.08.020 . 23993088 10.1016/j.cell.2013.08.020

[18] Martin M, Bell R, Bourgeois H, Brufsky A, Diel I, Eniu A, et al Bone-related complications and quality of life in advanced breast cancer: results from a randomized phase III trial of denosumab versus zoledronic acid. Clin Cancer Res. 2012;18(17):4841–9. doi: 10.1158/1078-0432.CCR-11-3310 . 22893628 10.1158/1078-0432.CCR-11-3310

[19] Holen I, Walker M, Nutter F, Fowles A, Evans CA, Eaton CL, et al Oestrogen receptor positive breast cancer metastasis to bone: inhibition by targeting the bone microenvironment in vivo. Clinical & experimental metastasis. 2016;33(3):211–24. doi: 10.1007/s10585-015-9770-x . 26585891 10.1007/s10585-015-9770-x

[20] Ottewell PD. The role of osteoblasts in bone metastasis. Journal of bone oncology. 2016;5(3):124–7. doi: 10.1016/j.jbo.2016.03.007 ; 27761372 10.1016/j.jbo.2016.03.007 PMC5063217

[21] Cox TR, Gartland A, Erler JT. Lysyl oxidase, a targetable secreted molecule involved in cancer metastasis. Cancer Res. 2016;76(2):188–92. doi: 10.1158/0008-5472.CAN-15-2306 . 26732355 10.1158/0008-5472.CAN-15-2306

[22] El-Haibi CP, Bell GW, Zhang J, Collmann AY, Wood D, Scherber CM, et al Critical role for lysyl oxidase in mesenchymal stem cell-driven breast cancer malignancy. Proc Natl Acad Sci U S A. 2012;109(43):17460–5. doi: 10.1073/pnas.1206653109 ; 23033492 10.1073/pnas.1206653109 PMC3491529

[23] Heaney RP. Vitamin D endocrine physiology. J Bone Miner Res. 2007;22 Suppl 2:V25–7. doi: 10.1359/jbmr.07s205 . 18290717 10.1359/jbmr.07s205

[24] Reginster JY. The high prevalence of inadequate serum vitamin D levels and implications for bone health. Curr Med Res Opin. 2005;21(4):579–86. doi: 10.1185/030079905X41435 . 15899107 10.1185/030079905X41435

[25] Lowry MB, Lotinun S, Leontovich AA, Zhang M, Maran A, Shogren KL, et al Osteitis fibrosa is mediated by Platelet-Derived Growth Factor-A via a phosphoinositide 3-kinase-dependent signaling pathway in a rat model for chronic hyperparathyroidism. Endocrinology. 2008;149(11):5735–46. doi: 10.1210/en.2008-0134 ; 18635661 10.1210/en.2008-0134 PMC2584582

[26] Wei EK, Wolin KY, Colditz GA. Time course of risk factors in cancer etiology and progression. J Clin Oncol. 2010;28(26):4052–7. doi: 10.1200/JCO.2009.26.9324 ; 20644083 10.1200/JCO.2009.26.9324 PMC4872328

[27] Rock CL. Milk and the risk and progression of cancer. Nestle Nutr Workshop Ser Pediatr Program. 2011;67:173–85. doi: 10.1159/000325583 . 21335998 10.1159/000325583

[28] Zheng Y, Zhou H, Modzelewski JR, Kalak R, Blair JM, Seibel MJ, et al Accelerated bone resorption, due to dietary calcium deficiency, promotes breast cancer tumor growth in bone. Cancer Res. 2007;67(19):9542–8. doi: 10.1158/0008-5472.CAN-07-1046 . 17909065 10.1158/0008-5472.CAN-07-1046

[29] James JJ, Evans AJ, Pinder SE, Gutteridge E, Cheung KL, Chan S, et al Bone metastases from breast carcinoma: histopathological—radiological correlations and prognostic features. Br J Cancer. 2003;89(4):660–5. doi: 10.1038/sj.bjc.6601198 ; 12915874 10.1038/sj.bjc.6601198 PMC2376918

[30] Rader JI, Baylink DJ, Hughes MR, Safilian EF, Haussler MR. Calcium and phosphorus deficiency in rats: effects on PTH and 1,25-dihydroxyvitamin D3. Am J Physiol. 1979;236(2):E118–22. . 420284 10.1152/ajpendo.1979.236.2.E118

[31] Hoey RP, Sanderson C, Iddon J, Brady G, Bundred NJ, Anderson NG. The parathyroid hormone-related protein receptor is expressed in breast cancer bone metastases and promotes autocrine proliferation in breast carcinoma cells. Br J Cancer. 2003;88(4):567–73. doi: 10.1038/sj.bjc.6600757 ; 12592371 10.1038/sj.bjc.6600757 PMC2377170

[32] Turner RT, Riggs BL, Spelsberg TC. Skeletal effects of estrogen. Endocr Rev. 1994;15(3):275–300. doi: 10.1210/edrv-15-3-275 . 8076582 10.1210/edrv-15-3-275

[33] Salemis NS. Skeletal muscle metastasis from breast cancer: management and literature review. Breast Dis. 2015;35(1):37–40. doi: 10.3233/BD-140384 . 25159186 10.3233/BD-140384

[34] Plaza JA, Perez-Montiel D, Mayerson J, Morrison C, Suster S. Metastases to soft tissue: a review of 118 cases over a 30-year period. Cancer. 2008;112(1):193–203. doi: 10.1002/cncr.23151 . 18040999 10.1002/cncr.23151

[35] Mathis S, Fromont-Hankard G, du Boisgueheneuc F, Godeneche G, Mahieu F, Balaboi I, et al [Muscular metastasis]. Rev Neurol (Paris). 2010;166(3):295–304. doi: 10.1016/j.neurol.2009.05.020 . 19732922 10.1016/j.neurol.2009.05.020

[36] Price J, Fabra A, Zhang R, Radinsky R, Pathak S. Characterization of variants of a human breast-cancer cell-line isolated from metastases in different organs of nude-mice. Int J Oncol. 1994;5(3):459–67. . 21559598 10.3892/ijo.5.3.459

