# Supplementary material for: Low calcium diet increases 4T1 mammary tumor carcinoma cell burden and bone pathology in mice
Source: PLoS One. 2017 Jul 27;12(7):e0180886. doi: 10.1371/journal.pone.0180886 (PMC5531562; doi:10.1371/journal.pone.0180886)
Supplement: S1 Table — (PDF) [file pone.0180886.s001.pdf]

**S1 Table**. Effects of Ca intake on cancellous and cortical bone architecture in contralteral (left; uninjected) proximal tibia metaphysis and distal tibia diaphysis, respectively in mice injected with 4T1 cells in right tibia and sacrificed on day 5, 10, and 21 post 4T1 cell injection.

| | Adequate Ca | | | Low Ca | | | FDR-adjusted Two-Way ANOVA P-values | | |
|---|---|---|---|---|---|---|---|---|---|
| | Day 5 | Day 10 | Day 21 | Day 5 | Day 10 | Day 21 | Ca Intake | Time | Interaction |
| **Proximal Tibia Metaphysis** (cancellous bone) | | | | | | | | | |
| Bone volume/tissue volume (%) | 25.8 ± 3.0 | 26.0 ± 1.7 | 20.2 ± 0.8 | 11.9 ± 1.3 | 13.4 ± 1.8 | 11.6 ± 1.0 | **0.000** | 0.247 | 0.608 |
| Trabecular number ($mm^{-1}$) | 7.1 ± 0.1 | 7.1 ± 0.1 | 7.0 ± 0.1 | 7.1 ± 0.1 | 7.2 ± 0.1 | 7.0 ± 0.1 | 0.639 | 0.639 | 0.908 |
| Trabecular thickness (µm) | 60 ± 2 | 61 ± 2 | 55 ± 1 | 51 ± 2 | 47 ± 2 | 47 ± 2 | **0.000** | 0.247 | 0.608 |
| Trabecular separation (µm) | 159 ± 3 | 157 ± 3 | 164 ± 3 | 172 ± 3 | 166 ± 3 | 169 ± 1 | **0.002** | 0.456 | 0.639 |
| **Distal Tibia Diaphysis** (cortical bone) | | | | | | | | | |
| Cross-sectional volume ($mm^3$) | 0.285 ± 0.008 | 0.284 ± 0.007 | 0.278 ± 0.006 | 0.280 ± 0.005 | 0.276 ± 0.005 | 0.285 ± 0.005 | 0.901 | 0.942 | 0.639 |
| Cortical volume ($mm^3$) | 0.238 ± 0.006 | 0.239 ± 0.004 | 0.233 ± 0.004 | 0.231 ± 0.004 | 0.225 ± 0.005 | 0.230 ± 0.003 | 0.132 | 0.920 | 0.639 |
| Marrow volume ($mm^3$) | 0.047 ± 0.003 | 0.045 ± 0.003 | 0.040 ± 0.002 | 0.050 ± 0.002 | 0.051 ± 0.002 | 0.054 ± 0.003 | **0.009** | 0.892 | 0.639 |
| Cortical thickness (µm) | 306 ± 5 | 308 ± 2 | 305 ± 3 | 293 ± 5 | 289 ± 5 | 288 ± 3 | **0.000** | 0.892 | 0.904 |
| Polar moment of inertia ($mm^4$) | 0.122 ± 0.007 | 0.122 ± 0.006 | 0.116 ± 0.005 | 0.118 ± 0.004 | 0.114 ± 0.005 | 0.122 ± 0.003 | 0.892 | 0.942 | 0.639 |

Data are mean ± SE, n=7-10/group
