# Supplementary material for: Low calcium diet increases 4T1 mammary tumor carcinoma cell burden and bone pathology in mice
Source: PLoS One. 2017 Jul 27;12(7):e0180886. doi: 10.1371/journal.pone.0180886 (PMC5531562; doi:10.1371/journal.pone.0180886)
Supplement: S2 Table — (PDF) [file pone.0180886.s002.pdf]

S2 Table. Effects of Ca intake and needle insertion on cancellous and cortical bone microarchitecture in tibia at 10 days post carrier injection.

	Adequate Ca		Low Ca		FDR-adjusted P-values		
					Paired test		Two-group Test
	Uninjected (left) Tibia	Carrier-injected (right) Tibia	Uninjected (left) Tibia	Carrier-injected (right) Tibia	Adequate Ca	Low Ca	Difference[a]
**Proximal Tibia Metaphysis** (cancellous bone)							
Bone volume/tissue volume (%)	29.8 ± 2.1	31.0 ± 4.0	12.9 ± 1.1	21.0 ± 2.1	0.888	**0.052**	0.792
Trabecular number (mm^{-1})	7.2 ± 0.1	7.4 ± 0.2	7.2 ± 0.1	7.5 ± 0.1	0.792	0.100	0.931
Trabecular thickness (µm)	65 ± 2	72 ± 3	50 ± 1	58 ± 2	0.313	0.081	0.895
Trabecular separation (µm)	154 ± 7	148 ± 7	170 ± 2	151 ± 3	0.895	**0.039**	0.792
**Distal Tibia Diaphysis** (cortical bone)							
Cross-sectional volume (mm^3)	0.282 ± 0.006	0.280 ± 0.003	0.285 ± 0.005	0.280 ± 0.004	0.844	0.863	0.895
Cortical volume (mm^3)	0.237 ± 0.005	0.230 ± 0.004	0.233 ± 0.004	0.230 ± 0.003	0.863	0.895	0.895
Marrow volume (mm^3)	0.046 ± 0.004	0.048 ± 0.004	0.053 ± 0.002	0.050 ± 0.001	0.863	0.728	0.731
Cortical thickness (µm)	304 ± 8	294 ± 8	294 ± 2	296 ± 2	0.792	0.863	0.792
Polar moment of inertia (mm^4)	0.121 ± 0.005	0.120 ± 0.003	0.122 ± 0.005	0.120 ± 0.003	0.844	0.900	0.931

Data are mean ± SE, n=6/group

[a]Interaction test of equal mean difference for the low and adequate calcium groups
