# Supplementary figures and images for: Low calcium diet increases 4T1 mammary tumor carcinoma cell burden and bone pathology in mice
Source: PLoS One. 2017 Jul 27;12(7):e0180886. doi: 10.1371/journal.pone.0180886 (PMC5531562; doi:10.1371/journal.pone.0180886)

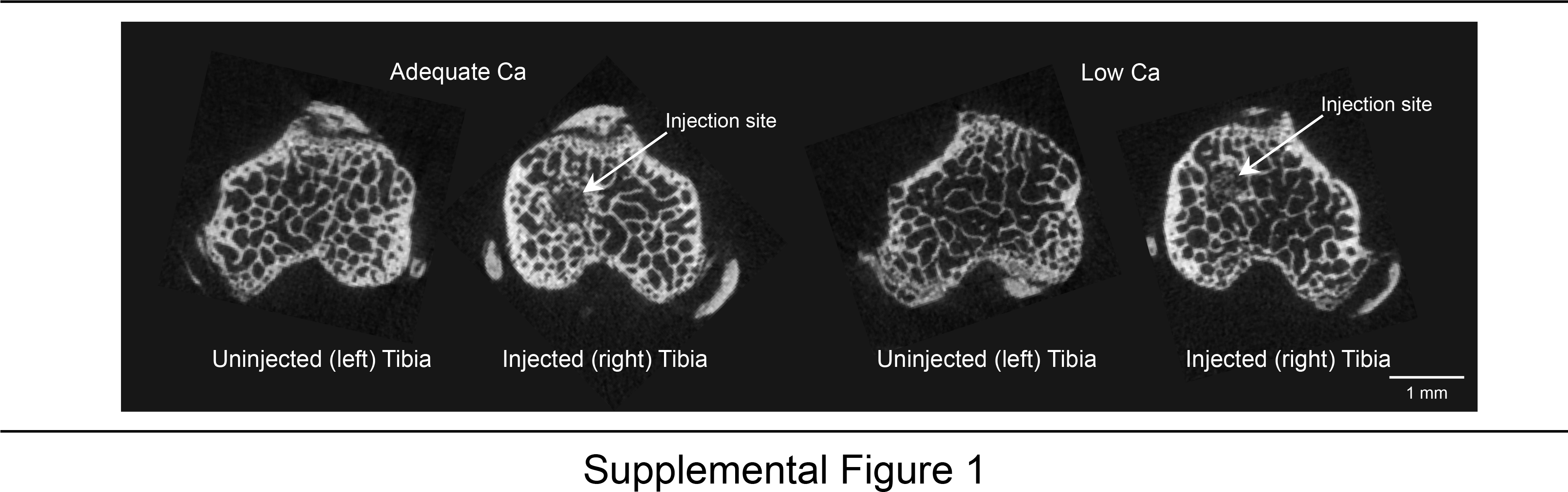

Supplement: S1 Fig — Note the presence of bone spicules in the needle track of both treatment groups. (TIF) [file pone.0180886.s003.tif]
